# Long-Term Preservation and Storage of Faecal Samples in Whatman^®^ Cards for PCR Detection and Genotyping of *Giardia duodenalis* and *Cryptosporidium hominis*

**DOI:** 10.3390/ani11051369

**Published:** 2021-05-12

**Authors:** Pamela Carolina Köster, Begoña Bailo, Alejandro Dashti, Carolina Hernández-Castro, Rafael Calero-Bernal, Francisco Ponce-Gordo, David González-Barrio, David Carmena

**Affiliations:** 1 Parasitology Reference and Research Laboratory, Spanish National Centre for Microbiology, Majadahonda, 28220 Madrid, Spain; pamelakster@yahoo.com (P.C.K.); BEGOBB@isciii.es (B.B.); dashti.alejandro@gmail.com (A.D.); carolina.hernandez1@udea.edu.co (C.H.-C.); 2 Group of Parasitology, Faculty of Medicine, Corporation of Tropical Pathologies, University of Antioquia, Medellín 050010, Colombia; 3 SALUVET, Department of Animal Health, Faculty of Veterinary, Complutense University of Madrid, 28040 Madrid, Spain; r.calero@ucm.es; 4 Department of Microbiology and Parasitology, Faculty of Pharmacy, Complutense University of Madrid, 28040 Madrid, Spain; pponce@ucm.es

**Keywords:** filter card, faeces, transportation, storage, preservation, *Giardia duodenalis*, *Cryptosporidium hominis*, PCR

## Abstract

**Simple Summary:**

Preservation and storage of biological samples prior to testing and analysis is a pressing issue in the epidemiological field studies conducted in remote or poor-resource areas with limited or no access to electricity where the cold chain cannot be maintained. This is particularly true for faecal specimens of human and animal origin exposed to high degradation rates under environmental conditions characterised by high temperatures and humidity, such as those present in tropical and subtropical regions. Under this scenario, simple, safe, and cost-effective methods are highly needed to allow the collection and transportation of well-preserved faecal samples intended for pathogen detection without compromising the performance, reliability, and accuracy of molecular procedures methods used for detection and genotyping purposes. This study assessed the suitability of three commercially available filter cards for the preservation of faecal samples containing common diarrhoea-causing enteric protozoan parasites at different storage periods and temperature conditions. Obtained results demonstrated that filter cards impregnated with faecal matrices containing these pathogens are fully compatible with downstream molecular methods for up to six months at room temperature. Therefore, filter cards can be used for the safe transportation, preservation, and storage of faecal samples without the need of the cold chain.

**Abstract:**

Preservation and conservation of biological specimens, including faecal samples, is a challenge in remote areas or poor-resource settings where the cold chain cannot be maintained. This study aims at evaluating the suitability of filter cards for long-term storage of faecal samples of animal and human origin positive to the diarrhoea-causing protozoan parasites, *Giardia duodenalis* and *Cryptosporidium hominis*. Three commercially available Whatman^®^ Filter Cards were comparatively assessed: the FTA^®^ Classic Card, the FTA^®^ Elute Micro Card, and the 903 Protein Saver Card. Human faecal samples positive to *G. duodenalis* (*n* = 5) and *C. hominis* (*n* = 5) were used to impregnate the selected cards at given storage (1 month, 3 months, and 6 months) periods and temperature (−20 °C, 4 °C, and room temperature) conditions. Parasite DNA was detected by PCR-based methods. Sensitivity assays and quality control procedures to assess suitability for genotyping purposes were conducted. Overall, all three Whatman^®^ cards were proven useful for the detection and molecular characterisation of *G. duodenalis* and *C. hominis* under the evaluated conditions. Whatman^®^ cards represent a simple, safe, and cost-effective option for the transportation, preservation, and storage of faecal samples without the need of the cold chain.

## 1. Introduction

Biological samples including blood, saliva, stools, urine, tissue, and cells have become increasingly valuable sources of genetic material for downstream DNA and RNA testing. Because PCR-based methods are extremely sensitive to the quality and purity of the starting nucleic acid material [1], the appropriate preservation of biospecimens during procurement, transportation, and storage is essential to maximise the success of laboratory analyses [2]. This is a pressing issue in field epidemiological studies conducted in non-clinical or remote locations where resources are scarce or lacking, or when the cold chain cannot be guaranteed. In these poor-resource settings, simple, safe, and cost-effective methods are highly needed to allow the collection and transportation of intact biospecimens without detrimental effect on their biophysical properties and diagnostic utility.

The use of solid supports, such as filter cards for the collection and analysis of biospecimens, began in 1961, when Robert Guthrie developed what is now known as the Guthrie Test by collecting drops of blood on filter paper for the detection of phenylketonuria in new-borns [3]. Since then, filter cards have been developed and commercialised by different companies [4], being the Whatman^®^ FTA^®^ card technology (Cytiva, Marlborough, MA, USA) one of the most frequently used. FTA^®^ cards are cotton-based, cellulose paper containing chemicals that lyse cells on contact, denature proteins, remove contaminants, and protect DNA from degradation (including UV radiation) by immobilising it onto the card’s matrix [5]. FTA^®^ cards allow the collection, preservation, and shipment of biospecimens for subsequent DNA and RNA analysis in a small space and at room temperature reducing transportation cost. Because chemically-treated FTA^®^ cards inactivate the pathogenic agents in the samples they are carrying, they do not usually require import/export permits. Some of them (e.g., the 903 Protein Saver Card) have been approved by the US Food and Drug Administration (FDA) as class 2 devices [6]. Whatman^®^ cards have been successfully used for the transportation and storage of a wide range of biospecimens including blood and serum [7], saliva [8], tissue [9], urine [10], sperm [11], mucus [12], and cerebral spinal fluid [13], among others.

Additionally, Whatman^®^ cards have been proven useful for the diagnosis of pathogenic blood parasites, including canine microfilariae [14] and malaria-causing *Plasmodium* species [15]. However, very few studies have attempted to evaluate their efficacy for the detection of enteric pathogens in stool samples [16,17,18]. The protozoan enteroparasites, *Giardia duodenalis* (syn. *G. intestinalis* and *G. lamblia*) and *Cryptosporidium* spp., are two of the major contributors to the global burden of diarrhoeal illness both in humans [19,20] and livestock [21,22] globally. In poor-resource settings, more than 200 million human cases of symptomatic giardiosis are reported annually [23], whereas cryptosporidiosis (primarily by *C. hominis* and *C. parvum*) is the second leading cause of diarrhoea and deaths (after rotavirus) in children younger than five years of age [24]. Remarkably, production (cattle, sheep, goats, horses, donkeys, Bactrian camels) and free-living (non-human primates, among others) animal species have all been demonstrated to be competent hosts for *C. hominis* globally, confirming that this *Cryptosporidium* species is indeed zoonotic [25].

This study aims at comparing the performance of three types of Whatman^®^ cards for the medium-/long-term preservation and storage (up to six months) of faecal material at different temperatures for downstream PCR detection of *G. duodenalis* and *Cryptosporidium hominis.*

## 2. Materials and Methods

### 2.1. Selected Whatman^®^ Cards

Three different commercially available Whatman^®^ cards with specific properties for sample collection, storage capacity, and costs were selected for comparative performance purposes: Whatman^®^ Classic Cards, FTA^®^ Elute Micro Cards, and 903 Protein Saver Cards (GE Healthcare Ltd., Cardiff, UK) (Table 1).

Whatman^®^ FTA^®^ Classic Cards contains chemical denaturants and a free radical scavenger that have the ability to lyse cells on contact, denature proteins, and protect DNA from degradation. The extracted DNA remains tightly bound to the matrix while cell membranes and organelles are lysed and proteins and inhibitors are washed away [26]. FTA^®^ Elute Micro Cards contains a chaotropic salt. Cells are lysed upon contact and proteins remain tightly bound while DNA is isolated from the matrix in a solution free of inhibitors with a simple water elution procedure [27]. Whatman^®^ 903 Protein saver Card is an untreated cotton fibber-based matrix. It does not stabilise nor protect DNA from degradation.

### 2.2. Stool Samples

Five fresh, independent stool samples of human origin with a positive result for *G. duodenalis* by real-time PCR (qPCR, see Section 2.6) with cycle threshold (Ct) values ranging from 29.0 to 34.3 (median: 31.3; standard deviation: 2.0) and confirmed as assemblage B, sub-assemblage BIV at the *gdh* locus by Sanger sequencing were selected for this study. Five fresh, independent stool samples of human origin with a positive result for *C. hominis* by nested small subunit ribosomal RNA (*ssu* rRNA)-PCR (see Section 2.7) and confirmed as *C. hominis* (genotype IbA10G2) by Sanger sequencing were also included (Table S1). Human samples were chosen by mere convenience in terms of accessibility and quantity, but the faecal material from non-human animal sources is equally valid.

Initial diagnosis and subsequent genotyping of the *G. duodenalis*- and *C. hominis*-positive stool samples were conducted at the Parasitology Reference and Research Laboratory of the Spanish National Centre for Microbiology (Majadahonda, Spain). After faecal sample homogenisation, 200 mg aliquots (*n* = 27, enough to cover all the experimental conditions considered in the study, see below) were weighed and stored at 4 °C in clean 1.5 mL Eppendorf tubes. Therefore, 270 stool sample aliquots were prepared.

### 2.3. Impregnation of Whatman^®^ Cards

**Figure 1:**
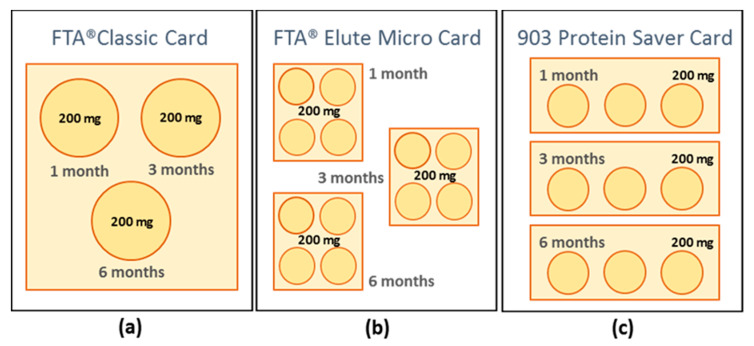
Standardisation of sampling areas for each Whatman^®^ card used in the present study to assess storage periods and conditions of impregnated stool samples (**a**) FTA^®^ Classic Card; (**b**) FTA^®^ Elute Micro Card; (**c**) 903 Protein Saver Card.

To standardise the experimental conditions of the study, total sample areas of the three Whatman*^®^* cards compared in the present study were normalised taking into consideration the sample area for each card specified in Table 1. A single sampling area of the FTA^®^ Classic Card (enough for impregnating 200 mg of faecal material, (Figure 1a) equalled to four sampling areas of the FTA^®^ Elute Micro Card (Figure 1b) and to three sampling areas of the 903 Protein Saver Card (Figure 1c). Under this premise, original Whatman^®^ cards were cut and rearranged, as shown in Figure 1, to allow the coverage of three (1 month, 3 months, and 6 months) storage periods. This arrangement was used in triplicate to test three (−20 °C, 4 °C, and room temperature) storage conditions. Room temperature was considered that in the range of 15 to 25 °C.

Normalised sampling units for each Whatman^®^ card were impregnated with 200 mg of each aliquoted stool sample described above using cotton swabs embedded in phosphate-buffered saline (PBS) to soften the faecal material and facilitate the impregnation process. Impregnated Whatman^®^ cards were allowed to dry at room temperature and stored in individual zip-lock plastic bags containing silica desiccant to keep moisture level low at the periods and storage conditions evaluated.

### 2.4. Sensitivity Assay

To estimate the minimum amount of *G. duodenalis* DNA detectable by qPCR in positive stool samples impregnated in Whatman*^®^* cards, one stool sample positive to this pathogen was selected. Serial-halved amounts (200 mg, 100 mg, 50 mg, 25 mg, 12.5 mg, and 6.25 mg) of faecal material were weighed and aliquoted in clean 1.5 mL Eppendorf tubes and subsequently used to impregnate single sampling areas of the Whatman*^®^* FTA*^®^* Classic Card. Impregnated cards were allowed to dry at room temperature and stored in individual zip-lock plastic bags containing silica desiccant for 1 month at room temperature. This experiment was not conducted with *Cryptosporidium*-positive samples because no semi-quantitative qPCR method was available in our laboratory for the detection of this pathogen.

### 2.5. DNA Extraction and Purification

Genomic DNA was extracted and purified from impregnated Whatman^®^ cards at each storage period and condition described in Section 2.3 and Section 2.4 using the QIAamp DNA Stool Mini Kit (QIAGEN^®^, Hilden, Germany), following the manufacturer’s instructions with minor modifications. Briefly, whole-normalised sampling surfaces of each compared Whatman^®^ card were cut into small pieces using a sterilised scissor and transferred into clean 2 mL Eppendorf tubes containing 1 mL of Inhibitex buffer. After incubation at 95 °C for 10 min, the tubes were thoroughly vortexed and centrifuged at 13,000 rpm for 3 min. Then, 350 µL of the obtained supernatants were transferred to clean 1.5 mL Eppendorf tubes and the rest of the procedure was completed using the QIAcube (QIAGEN^®^) automated DNA extraction system. Purified genomic DNA (200 µL) was stored at 4 °C until downstream PCR testing.

### 2.6. Molecular Detection and Characterisation of Giardia duodenalis

Detection of *G. duodenalis* DNA was achieved using a real-time PCR (qPCR) method targeting a 62-bp region of the *ssu* rRNA) gene of the parasite, as described elsewhere [28]. Amplification reactions (25 µL) contained 3 µL of template DNA, 12.5 pmol of primers Gd-80F and Gd-127R, 10 pmol of probe, and 12.5 μL TaqMan^®^ Gene Expression Master Mix (Applied Biosystems, California, CA, USA). Detection of parasitic DNA was performed on a Corbett Rotor GeneTM 6000 real-time PCR system (QIAGEN^®^). Water (no template) and genomic DNA (positive) controls were included in each PCR run.

For genotyping purposes, a semi-nested PCR was used to amplify a 432-bp fragment of the glutamate dehydrogenase (*gdh*) of *G. duodenalis* [29]. Briefly, PCR reaction mixtures (25 μL) included 5 μL of template DNA and 0.5 μM of the primer pairs GDHeF/GDHiR in the primary reaction and GDHiF/GDHiR in the secondary reaction.

### 2.7. Molecular Detection and Characterisation of Cryptosporidium hominis

Detection of *C. hominis* DNA was achieved using a nested-PCR protocol to amplify a 587-bp fragment of the *ssu* rRNA gene of the parasite as described elsewhere [30]. Amplification reactions (50 μL) included 3 μL of DNA sample and 0.3 μM of the primer pairs CR-P1/CR-P2 in the primary reaction and CR-P3/CPB-DIAGR in the secondary reaction. Reaction mixes also contained 2.5 units of MyTAQ™ DNA polymerase (Bioline GmbH, Luckenwalde, Germany) and 5× MyTAQ™ Reaction Buffer containing 5 mM dNTPs and 15 mM MgCl_2_.

For genotyping purposes, a nested PCR was used to amplify an 870-bp fragment of the 60 kDa glycoprotein (*gp60*) of *C. hominis* [31]. PCR reaction mixtures (50 μL) included 2‒3 μL of template DNA and 0.3 μM of the primer pairs AL-3531/AL-3535 in the primary reaction and AL-3532/AL-3534 in the secondary reaction.

The semi-nested and nested PCR protocols described above were conducted on a 2720 Thermal Cycler (Applied Biosystems). Reaction mixes included 2.5 units of MyTAQ™ DNA polymerase (Bioline GmbH, Luckenwalde, Germany), and 5× MyTAQ™ Reaction Buffer containing 5 mM dNTPs and 15 mM MgCl_2_. Amplicons were visualised under UV light after 2% agarose gel electrophoresis. Positive-PCR products were directly sequenced in both directions using inner primer sets. DNA sequencing was conducted by capillary electrophoresis using the BigDye^®^ Terminator chemistry (Applied Biosystems) on an on ABI PRISM 3130 automated DNA sequencer.

### 2.8. Quality Control

To confirm the suitability of the three compared Whatman^®^ cards for genotyping purposes, purified genomic DNAs from single *G. duodenalis*- and *C. hominis*-positive samples stored for six months (the maximum period covered in the present study) at 4 °C and room temperature (the most sensitive conditions to DNA damage evaluated here) were re-amplified by *gdh*-PCR (*G. duodenalis*) and *gp60*-PCR (*C. hominis*) and sequenced as described above. The quality of the obtained chromatograms was visually inspected, and the accuracy of the readings confirmed by alignment with appropriate reference sequences retrieved from GenBank.

### 2.9. Statistical Analyses

The Shapiro–Wilk’s test was used to assess the normality of distribution of the Ct values obtained in *G. duodenalis*-positive samples during qPCR analyses at each period and storage condition evaluated. Once normality was demonstrated, an analysis of variance (ANOVA) for simultaneous comparison of conditions was conducted. A probability (*p*) value < 0.05 was considered evidence of statistical significance. Statistical analyses were performed using the R-software version 4.0.2 [32].

## 3. Results

### 3.1. Performance of Whatman^®^ Cards for the Preservation and Storage of G. duodenalis-Positive Faecal Samples

**Figure 2:**
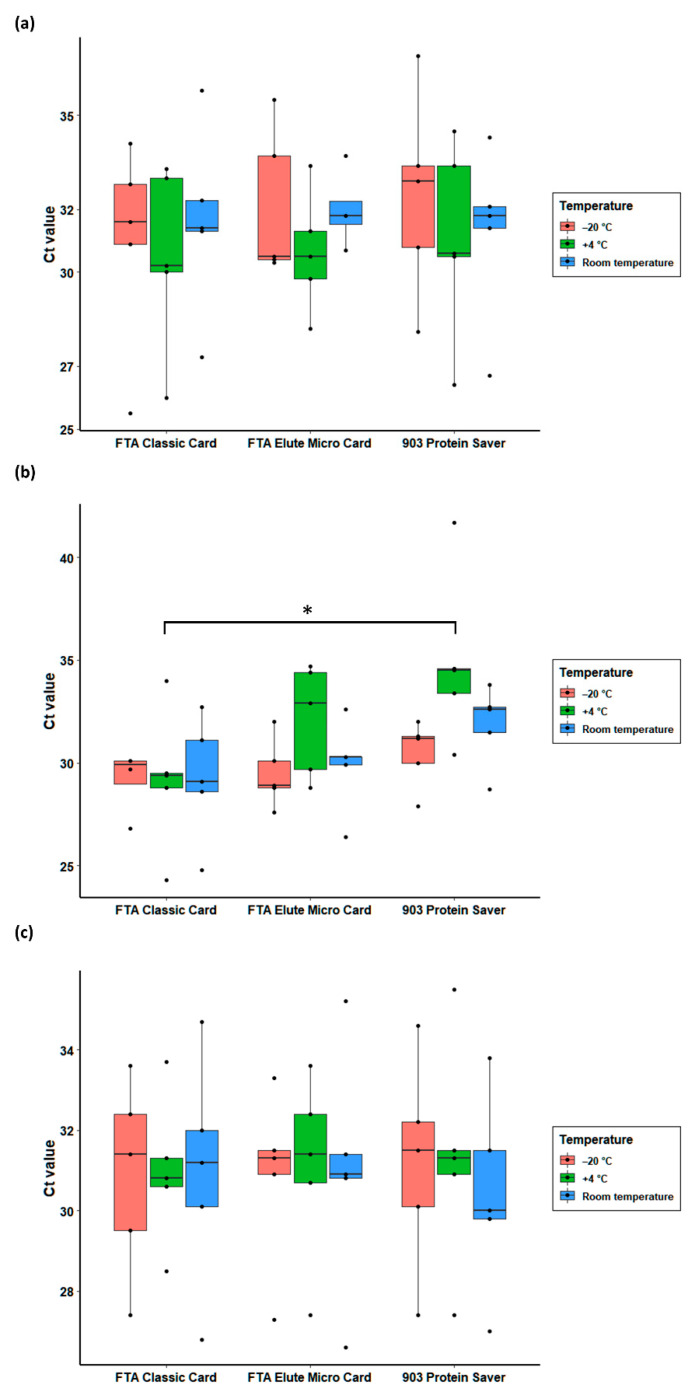
Box plot of cycle threshold (Ct) values generated from *Giardia duodenalis* isolates extracted from the three Whatman^®^ cards evaluated in the present study at different storage conditions. (**a**): 1 month-length storage; (**b**): 3 months-length storage; (**c**): 6 month-length storage. Horizontal thick lines within boxes represent median values Upper and lower whiskers represent the data range. Plotted dots represent outliers. Using the Tukey’s Honestly Significant Difference test as multiple post hoc comparison method, statistical significance is represented as * (*p* < 0.05).

All except two samples (an FTA*^®^* Elute Micro Card stored at room temperature for 1 month, and an FTA*^®^* Classic Card stored at ‒20 °C for three months) tested positive for *G. duodenalis* by qPCR, yielding Ct values similar to those generated at the time of initial diagnosis (Table S1). At 1 month-length storage, all three Whatman*^®^* cards performed equally well, with those kept at 4 °C yielding lower (but not statistically significant, *p* = 0.96) Ct values (Figure 2a). At 3 months-length storage, the FTA*^®^* Classic Card provided the best diagnostic values in terms of sensitivity (lower Ct values) and precision (lower standard deviation) regardless of the temperature. These results were statistically significant when compared with those obtained with the 903 Protein Saver Card (*p* = 0.01), but not with the FTA*^®^* Elute Micro Card (*p* = 0.40) (Figure 2b). No statistically significant differences (*p* = 0.99) were observed among the three compared Whatman*^®^* cards at 6 months-length storage (Figure 2c). It should be noted that the difference observed between the FTA*^®^* Classic Card and the 903 Protein Saver Card in Figure 2b is associated to the effect caused by an outlier data value generated with the latter at 4 °C storage conditions. Removal of this value resulted in the loss of statistical significance.

### 3.2. Performance of Whatman^®^ Cards for the Preservation and Storage of C. hominis-Positive Faecal Samples

**Figure 3:**
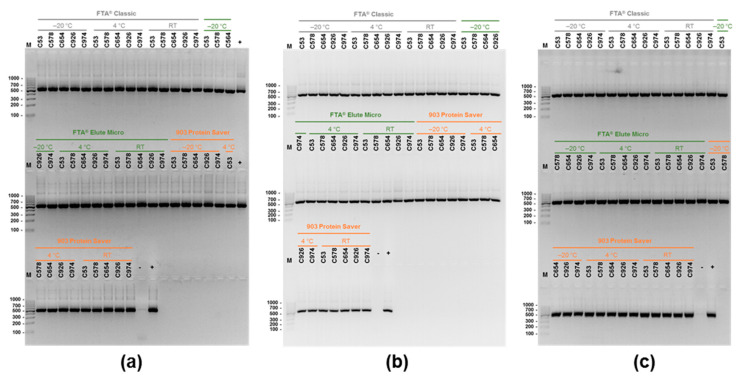
Agarose gel electrophoresis (2% *w*/*v*) detection of PCR products showing the presence of a 587-bp amplicon for the *Cryptosporidium hominis ssu* rRNA gene extracted from the three Whatman^®^ cards evaluated in the present study at different storage conditions. (**a**): 1 month-length storage; (**b**): 3 months-length storage; (**c**): 6 month-length storage.

The generated *ssu*-PCR results after the processing and testing of *C. hominis*-positive stool samples impregnated in the Whatman*^®^* cards at the storage periods and conditions assessed in the present study are shown in Figure 3. All tested samples stored for 1 month (Figure 3a), 3 months (Figure 3b), or 6 months (Figure 3c) yielded clear amplicons regardless of the storage temperature considered (Figure S1).

### 3.3. Sensitivity Assay

When tested by qPCR, serial-halved amounts of faecal material containing *G. duodenalis* cysts impregnated in Whatman^®^ FTA^®^ Classic Cards generated Ct values ranging from 25.6 (corresponding to 200 mg of faeces) to 33.1 (corresponding to 6.3 mg of faeces) (Table 2).

### 3.4. Quality Control

Figure S1 shows the *gdh*-PCR and *gp60*-PCR amplification results obtained with the purified genomic DNAs from the two samples positive to *G. duodenalis* and *C. hominis*, respectively. Both samples were stored in all three compared Whatman^®^ cards for 6 months (the maximum period covered in the present study) at 4 °C and room temperature (the most likely conditions to induce DNA damage evaluated here). Two of the six *G. duodenalis*-positive extracts were successfully amplified at the *gdh* locus (Figure S1a) and their associated chromatograms displayed good quality sequences confirming the identity (sub-assemblage BIV) of the parasite (Figure S2a). All *C. hominis*-positive extracts yielded clear amplicons at the *gp60* locus, irrespectively of the Whatman^®^ card used or the temperature considered (Figure S1b). Sanger sequencing analysis revealed good quality sequence data confirming the identity (genotype IbA10G2) of the parasite (Figure S2b).

## 4. Discussion

This study evaluated the suitability of three commercially available Whatman^®^ Filter Cards (the FTA^®^ Classic Card, the FTA^®^ Elute Micro Card, and the 903 Protein Saver Card) for the long-term storage of faecal material containing *G. duodenalis* cysts and *C. hominis* oocysts, two of the major contributors to the global burden of diarrhoeal illness both in humans [19,20] and livestock [21,22] globally. Of note, both protozoan parasites present aggregated distributions depending on the host species, genetic variants, or even geographical area considered [33,34,35,36,37]. Because both *G. duodenalis* and *Cryptosporidium* spp. are common findings in the faecal material of human and animal hosts, some of their species/genotypes have zoonotic potential, and are ubiquitous in the environment, research on the epidemiology and transmission of these pathogens should be always conducted under the One Health umbrella. This approach is particularly useful in those epidemiological scenarios where different epidemiological (e.g., domestic and sylvatic) cycles of the parasites overlap, allowing the occurrence of spillover events [33,34,36]. The three major contributions of this survey include the demonstration that (i) the three compared Whatman^©^ cards performed near equally well in maintaining the stability of the faecal material for up to six months irrespectively of the storage temperature; (ii) the parasitic DNA extracted from impregnated Whatman^®^ cards was suitable for subsequent molecular detection and genotyping purposes; and (iii) Whatman^®^ cards represent simple, time- and cost-effective options for the safe storage and transportation of faecal samples of human and animal origin without the need of the cold chain.

FTA^®^ card technology was originally designed as a matrix for blood storage and processing medium [5]. Because of their versatility and simplicity of use, FTA^®^ cards were soon after tested for storing other biological samples including saliva [8], tissue [9], urine [10], sperm [11], mucus [12], and cerebral spinal fluid [13]. This tool has been also assessed for the molecular detection of gastrointestinal parasites (e.g., the coccidian *Cryptosporidium* spp. and *Cyclospora cayetanensis*, the flagellated *G. duodenalis*, and the microsporidia *Encephalitozoon intestinalis*) in matrices including clinical specimens and fresh produce [16,17,18]. In a seminal study, FTA^®^ card templates prepared from purified *Cryptosporidium* spp. oocysts and *E. intestinalis* spores and subsequently assessed by PCR allowed the identification of as few as 10 oocysts/spores. Similar results were also observed with clinical samples including faeces, urine, sputum, and foods (berries) [16]. The authors concluded that PCR analysis using the FTA^®^ card format for DNA template preparation was routinely unaffected by the matrix from which the sample was derived while still maintaining a high level of detection sensitivity [16]. In a subsequent survey, known concentrations of *G. duodenalis* cysts and *Cryptosporidium* spp. oocysts were serially diluted, spiked into a faecal suspension from a pathogen-negative stool, and smeared onto FTA^®^ Elute Micro cards [17]. Stool cards were then stored at room temperature, DNA was extracted and purified using QIAGEN protocols and tested by multiplex PCR coupled with Luminex assay at 1 week, 1 month, and 3 months. A limited number of stool cards were also stored at 4 °C and in a humid incubator at 31 °C for 1 week to determine the impact of environmental conditions on detection. The authors detected *G. duodenalis* at 3 months with a 2-log reduction from the original concentration, whereas *Cryptosporidium* spp. was undetected after 1 month of storage. Failure to detect the presence of *Cryptosporidium* spp. for longer periods of time was attributed to suboptimal breakage of the parasite oocyst wall [17]. Finally, the 903 Protein Saver card has been evaluated for the detection of *G. duodenalis*, *Cryptosporidium* spp., and *Entamoeba histolytica* in either whole faecal samples or stool suspensions using QIAGEN procedures for DNA purification and qPCR for detection [18]. In this study the cards were stored for only 48 h before DNA purification and qPCR testing. Depending on the starting (whole or suspension) faecal material used for impregnation, obtained overall sensitivities were 85–95% for *G. duodenalis*, 60–85% for *E. histolytica*, and 35–40% for *Cryptosporidium* spp. In general, faecal suspensions yielded poorest qPCR amplification results than whole faecal samples. Parasite load was identified as a critical factor for qPCR success [18].

This study improves current knowledge on the practicality and performance of Whatman^®^ cards for the molecular detection of diarrhoea-causing enteric protozoan parasites in several aspects. First, this is (to author´s knowledge) the first attempt conducted to date to compare simultaneously three different types of Whatman^®^ cards including the FTA^®^ Classic Card, the FTA^®^ Elute Micro Card, and the 903 Protein Saver Card. Previous studies focused on a specific card type only [16,17,18]. Second, the evaluated storage period has been extended to 6 months, three more months that the maximum period covered in previous studies [17]. Third, this survey evaluated the effect of three different storage temperatures. Freezing (–20 °C) and refrigeration (4 °C) temperatures represented the most common conditions in routine laboratory practice, whereas the room temperature condition attempted to mimic those present in field work characterised by lack of electric supply where sample conservation is a pressing issue. Previous studies were conducted primarily at room temperature [16,17,18], with only few impregnated cards being tested at other temperatures [17]. Fourth, present results were obtained exclusively with true clinical faecal samples, whereas those from previous studies were mostly derived from purified parasitic material [16] or artificially spiked stools [17]. Fifth, quality control data presented here provided evidence demonstrating that Whatman^®^ cards were suitable for genotyping (in addition to detection) purposes, including Sanger sequencing. None of the studies carried out before assessed this possibility.

A major contribution of this study was the finding that all three compared Whatman^®^ cards yielded sensitivity values near 100%, irrespectively of the storage period, the temperature considered, or the parasite species investigated. These figures were considerably higher than those reported in similar surveys [16,17,18]. Several factors may account, at least partially, for the differences observed. For instance, we used high impregnation loads (200 mg) of faecal material, in line with the recommendations of the QIAGEN procedure used for DNA extraction and purification. In addition, our protocol included a modification (sample incubation with Inhibitex buffer at 95 °C for 10 min) specifically intended at improving the efficiency of cyst/oocyst breakage, an issue previously identified as a factor limiting the diagnostic sensitivity of PCR assays [17]. Variations in the diagnostic performance of the PCR methods used (conventional nested PCR, qPCR, multiplex qPCR) may also influence the amplification success rate obtained in these studies.

This study presents, however, some limitations that should be taken into consideration. For instance, a straightforward application of the data presented here is the potential usefulness of Whatman^®^ cards as a convenient stool storage system for periods longer than 6 months (e.g., in biobanks), a possibility that should be conveniently evaluated in further studies. Of note, room temperature was considered here those in the range of 15 to 25 °C. More extreme temperature (and humidity) conditions, such as those typically present in tropical and sub-tropical regions, may affect the performance of the Whatman^®^ cards. This possibility should be conveniently evaluated in future studies. Also, only faecal samples positive for *G. duodenalis* and *C. hominis* were investigated. Although we do not anticipate significant performance differences with other enteric protist species, this is also an issue that remains to be fully elucidated. Finally, our data can be used as proof of concept for the suitability of Whatman^®^ technology for the safe storage and transportation of faecal material at room temperature without detrimental effects on stability and diagnostic features, although this fact should be demonstrated in *ad*-*hoc* studies.

## 5. Conclusions

Data presented here demonstrate that Whatman^®^ cards are a cost-effective option for the preservation and long-term storage (up to six months) of faecal samples under a wide range of temperatures (from –20 °C to room temperature) without compromising their biospecimen stability and suitability for molecular-based diagnostic methods. Indeed, Whatman^®^ cards enable the molecular detection and genotyping of common diarrhoea-causing enteric protozoan parasites, including *C. hominis* and *G. duodenalis*. Further research should be conducted to unambiguously demonstrate the usefulness of Whatman^®^ cards in field epidemiological surveys involving larger number of faecal samples, wider ranges of temperature and humidity conditions, and storage periods longer than six months. In practical terms, Whatman^®^ cards would allow the obtaining and safe transportation of faecal samples of human and animal origin from remote areas to clinical or research laboratories without the need of the cold chain.

## Figures and Tables

**Table 1:** Main features of the Whatman^®^ cards used in the present comparative study.

Card	Reference	Cards/Pack	Sample Areas/ Card	Total Volume/Card (µL)	Total Sample Area (cm^2^)/Card
FTA^®^ Classic	WB120205	100	4	500	19.6
FTA^®^ Elute Micro	WB120410	100	4	120	4.0
903 Protein Saver	10531018	100	5	390‒400	6.1

**Table 2:** Cycle threshold (Ct) values obtained by real-time PCR in serially-halved amounts of a faecal sample positive for *G. duodenalis* impregnated in Whatman^®^ FTA^®^ Classic Cards.

Faecal Material (mg)	Ct Value
200	25.6
100	27.3
50	27.1
25	28.7
12.5	30.2
6.25	33.1

## Data Availability

All relevant data are within the article and its additional files.

## Supplementary Materials

The following are available online at https://www.mdpi.com/article/10.3390/ani11051369/s1, Table S1: Real-time PCR cycle threshold values obtained after the amplification of *Giardia duodenalis*-positive stool samples impregnated in the Whatman^®^ Cards at the storage periods and conditions assessed in the present study, Figure S1: Agarose gel electrophoresis (2% *w*/*v*) detection of PCR products used for evaluating the suitability of Whatman^®^ cards for genotyping and Sanger sequencing purposes. (a) Results showing the presence of a 432-bp amplicon for the *Giardia duodenalis gdh* gene in sample G145. Some lanes have been cut and re-arranged to keep the same order in the whole figure; (b): Results showing the presence of an 870-bp amplicon for the *Cryptosporidium hominis gp60* gene in sample C578., Figure S2: Representative chromatograms showing Sanger sequencing results for PCR amplicons generated from genomic DNA extracted and purified from Whatman^®^ Cards. (a): Results for the *Giardia duodenalis gdh* gene in sample G145; (b): Results for the *Cryptosporidium hominis gp60* gene in sample C578.
