# Supplementary material for: Long-Term Preservation and Storage of Faecal Samples in Whatman® Cards for PCR Detection and Genotyping of Giardia duodenalis and Cryptosporidium hominis
Source: Animals (Basel). 2021 May 12;11(5):1369. doi: 10.3390/ani11051369 (PMC8151430; doi:10.3390/ani11051369)
Supplement: Supplementary file 1 [file animals-11-01369-s001.zip › Figure S1.pdf]

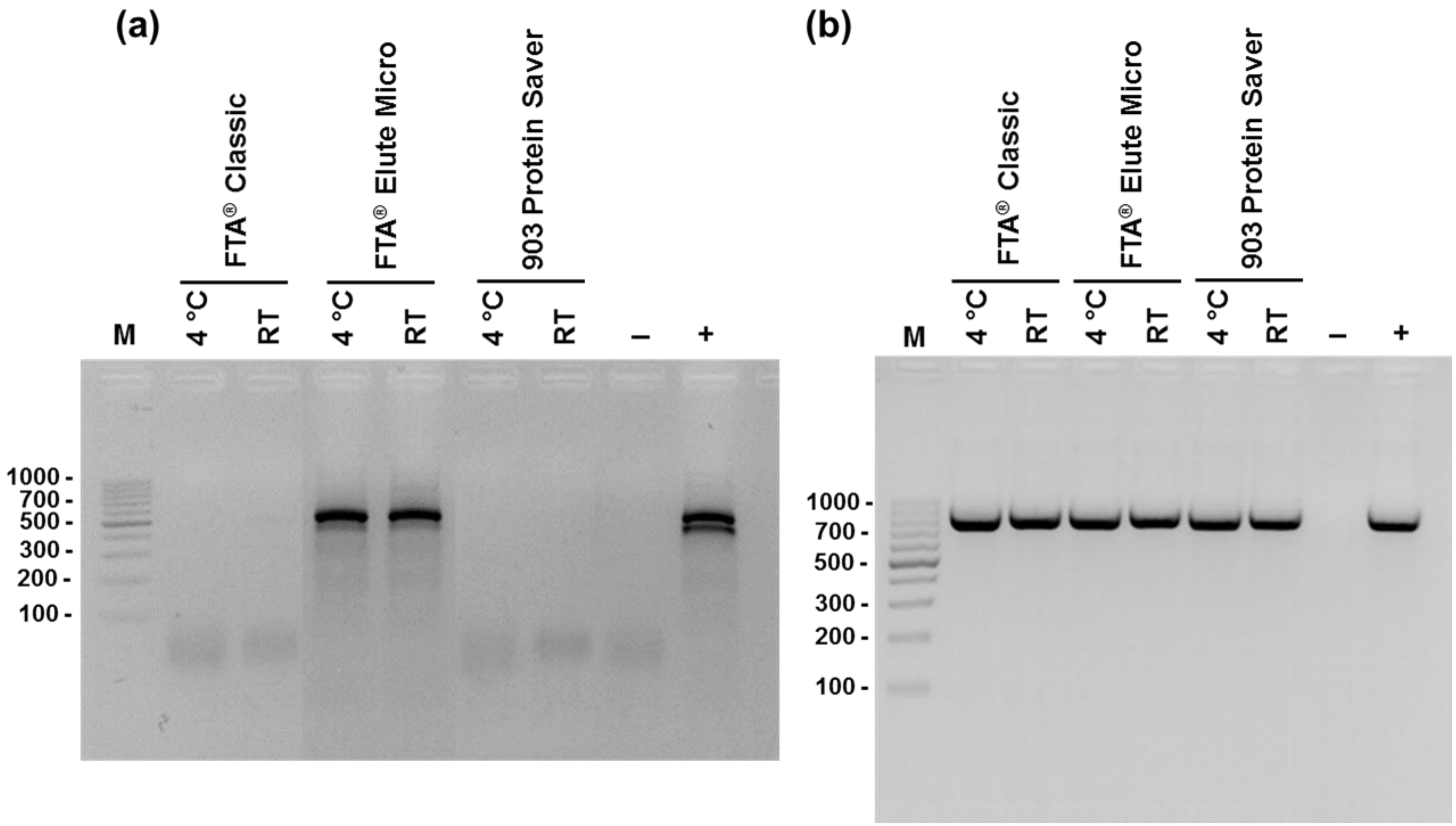

Figure S1. Agarose gel electrophoresis (2% w/v) detection of PCR products used for evaluating the suitability of Whatman® cards for genotypying and Sanger sequencing purposes. (**a**) Results showing the presence of a 432-bp amplicon for the *Giardia duodenalis gdh* gene in sample G145. Some lanes have been cut and re-arranged to keep the same order in the whole figure; (**b**): Results showing the presence of a 870-bp amplicon for the *Cryptosporidium hominis gp60* gene in sample C578.
