# Supplementary material for: Long-Term Preservation and Storage of Faecal Samples in Whatman® Cards for PCR Detection and Genotyping of Giardia duodenalis and Cryptosporidium hominis
Source: Animals (Basel). 2021 May 12;11(5):1369. doi: 10.3390/ani11051369 (PMC8151430; doi:10.3390/ani11051369)

animals

MDPI

Figure S2. Representative chromatograms showing Sanger sequencing results for PCR amplicons generated from genomic DNA extracted and purified from Whatman® cards. (**a**): Results for the *Giardia duodenalis gdh* gene in sample G415; (**b**): Results for the *Cryptosporidium hominis gp60* gene in sample C578. For each sample, forward and reverse sequences are shown.

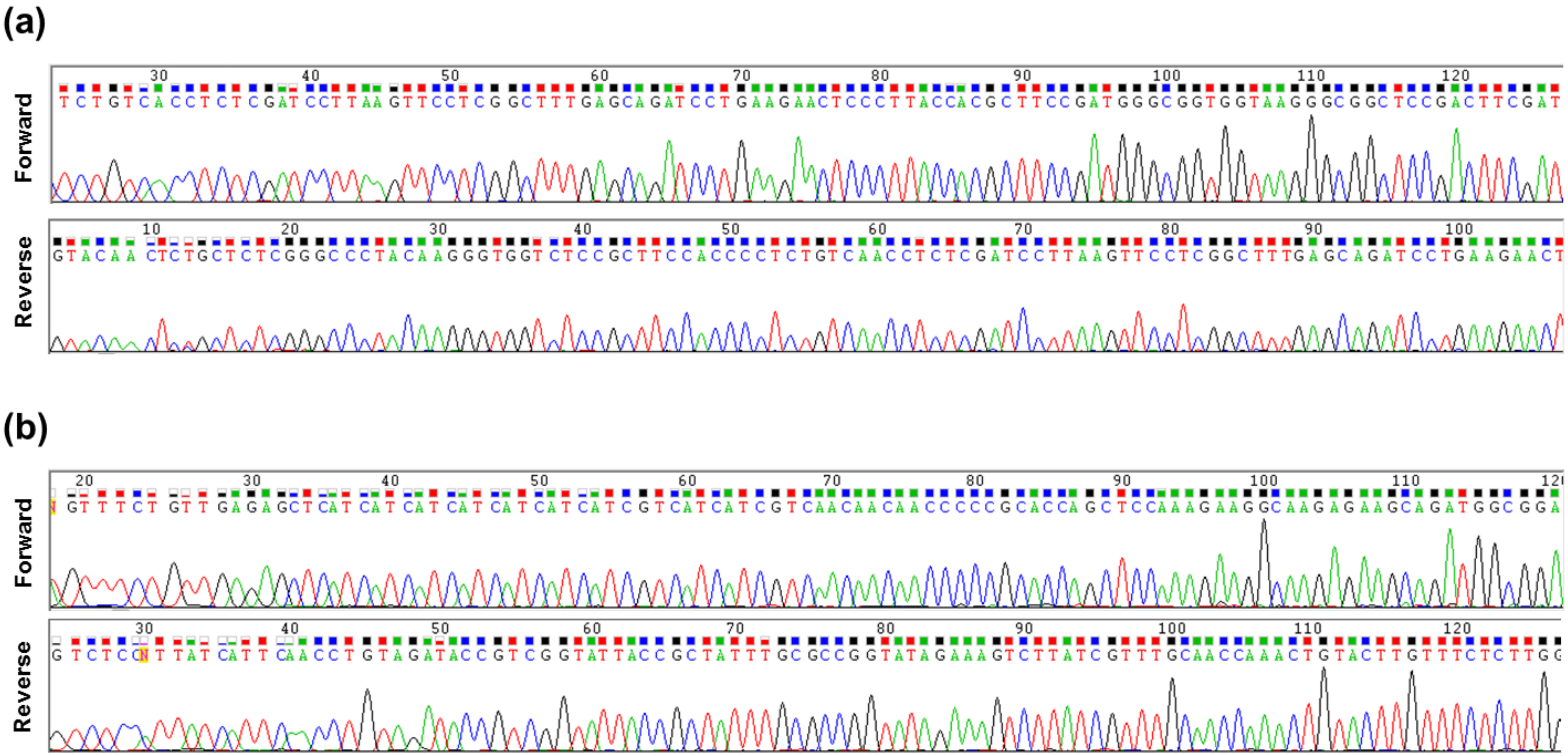

Supplement: Supplementary file 1 [file animals-11-01369-s001.zip › Figure S2.pdf]
