# Supplementary material for: Long-Term Preservation and Storage of Faecal Samples in Whatman® Cards for PCR Detection and Genotyping of Giardia duodenalis and Cryptosporidium hominis
Source: Animals (Basel). 2021 May 12;11(5):1369. doi: 10.3390/ani11051369 (PMC8151430; doi:10.3390/ani11051369)
Supplement: Supplementary file 1 [file animals-11-01369-s001.zip › Table S1.pdf]

animals

MDPI

Table S1. Real-time PCR cycle threshold values obtained after the amplification of *Giardia duodenalis*-positive stool samples impregnated in the Whatman® Cards at the storage periods and conditions assessed in the present study.

			1 Month			3 Months			6 Months		
**Whatman® Card**	**Sample**	**Ct[1] value**	**−20 °C**	**+4 °C**	**RT**	**−20 °C**	**+4 °C**	**RT**	**−20 °C**	**+4 °C**	**RT**
FTA® Classic Card	G134	32.4	32.8	33.0	32.3	30.1	29.4	31.1	32.4	30.6	32.0
	G145	29.0	25.5	26.0	27.3	26.8	24.3	24.8	27.4	28.5	26.8
	G249	34.3	34.1	33.3	35.8	Neg.[2]	34.0	32.7	33.6	33.7	34.7
	G351	30.6	31.6	30.2	31.4	29.7	29.5	29.1	29.5	31.3	30.1
	G353	31.3	30.9	30.0	31.3	30.1	28.8	28.6	31.4	30.8	31.2
FTA® Elute Micro Card	G134	32.4	33.7	29.8	30.7	30.1	32.9	30.3	31.3	30.7	30.8
	G145	29.0	30.3	28.2	Neg.[2]	27.6	28.8	26.4	27.3	27.4	26.6
	G249	34.3	35.5	33.4	33.7	32.0	34.4	32.6	33.3	33.6	35.2
	G351	30.6	30.5	31.3	31.8	28.8	34.7	30.3	31.5	31.4	30.9
	G353	31.3	30.4	30.5	31.8	28.9	29.7	29.9	30.9	32.4	31.4
903 Protein Saver Card	G134	32.4	33.4	33.4	32.1	30.0	34.6	32.7	32.2	31.5	31.5
	G145	29.0	28.1	26.4	26.7	27.9	30.4	28.7	27.4	27.4	27.0
	G249	34.3	36.9	34.5	34.3	31.3	41.7	33.8	34.6	35.5	33.8
	G351	30.6	32.9	30.6	31.4	32.0	34.5	31.5	31.5	30.9	30.0
	G353	31.3	30.8	30.5	31.8	31.2	33.4	32.6	30.1	31.3	29.8

[1] Ct: cycle threshold value obtained at the time of diagnosis.
[2] Sample tested twice to confirm the negative result.
